# Supplementary material for: Oligonucleotide Capture Sequencing of the SARS-CoV-2 Genome and Subgenomic Fragments from COVID-19 Individuals
Source: bioRxiv. 2020 Dec 11:2020.12.11.421057. Preprint. [Version 1] doi: 10.1101/2020.12.11.421057 (PMC7743067; doi:10.1101/2020.12.11.421057)
Supplement: 1 [file NIHPP2020.12.11.421057-supplement-1.pdf]

bioRxiv preprint doi: https://doi.org/10.1101/2020.12.11.421057; this version posted December 11, 2020. The copyright holder for this preprint (which was not certified by peer review) is the author/funder, who has granted bioRxiv a license to display the preprint in perpetuity. It is made available under aCC-BY 4.0 International license.

Supporting information

S1 Fig. Genome coverage plots for the three SARS-CoV-2 negative samples. Coverage is localized despite the 45-91 M reads that these samples obtained post-capture.

S2 Fig. Genome coverage plots. Genome coordinates on X-axis and coverage in log scale of Y-axis for the 17 samples with full length SARS-CoV-2 genome reconstructions

S3 Fig. A multiple sequence alignment (using MAFFT) of 17 reconstructed SARS-CoV-2 genomes and Wuhan-Hu-1 reference genome (NC_045512). Grey indicates agreement with the reference, black is a disagreement, and pink marks areas in the reconstruction with an ambiguous nucleotide, "N". The pangolin lineage assignment is listed next to the sample name. The extra length of the 192000251B seen here is an assembly artifact and was excluded from analysis.

S4 Fig. Stop codon variants in sampled SARS-CoV-2 genomic assemblies. A snapshot of full length SARS-CoV-2 genome assemblies from GISAID and NCBI on 27 May 2020 was downloaded (comprising 39246 entries), and processed to detect single nucleotide variant alleles that introduced a stop codon. Introduced stop codons were detected in 270 entries, and the frequency of these alleles are plotted along the SARS-CoV-2 reference genome position. Introduced stop codons are rare but are distributed throughout the genomic sequences. Multiple loci harbor stop codons in unrelated assemblies.

S5 Fig. Junctions reads to support expression of ORF10 192000052B, 192000251B and 192000440B. Expression values were calculated as 0.13, 0.13 and 0.02 reads/million. Few examples of those junction reads are shown in the figure (purple arrows).

bioRxiv preprint doi: https://doi.org/10.1101/2020.12.11.421057; this version posted December 11, 2020. The copyright holder for this preprint (which was not certified by peer review) is the author/funder, who has granted bioRxiv a license to display the preprint in perpetuity. It is made available under aCC-BY 4.0 International license.

S1 Table. Sample information, capture pools and sequencing metrics details.

S2 Table. Lineage analysis of the 17 full-length genomes.

S3 Table. Junction read counts is reads/million identified in the post capture data of 17 samples with full-length genomes.

S4 Table. Junction read counts in reads/million identified in the nine samples sequenced before (IDxxxxB-2) and after capture (IDxxxxB) enrichment.
